# New clues in the nucleus: transcriptional reprogramming in effector-triggered immunity

**DOI:** 10.3389/fpls.2013.00364

**Published:** 2013-09-13

**Authors:** Saikat Bhattacharjee, Christopher M. Garner, Walter Gassmann

**Affiliations:** ^1^Division of Plant Sciences, University of MissouriColumbia, MO, USA; ^2^Christopher S. Bond Life Sciences Center and Interdisciplinary Plant Group, University of MissouriColumbia, MO, USA; ^3^Division of Biological Sciences, University of MissouriColumbia, MO, USA

**Keywords:** effector-triggered immunity, transcriptional reprogramming, transcription factors, avirulence genes, resistance proteins

## Abstract

The robustness of plant effector-triggered immunity is correlated with massive alterations of the host transcriptome. Yet the molecular mechanisms that cause and underlie this reprogramming remain obscure. Here we will review recent advances in deciphering nuclear functions of plant immune receptors and of associated proteins. Important open questions remain, such as the identities of the primary transcription factors involved in control of effector-triggered immune responses, and indeed whether this can be generalized or whether particular effector-resistance protein interactions impinge on distinct sectors in the transcriptional response web. Multiple lines of evidence have implicated WRKY transcription factors at the core of responses to microbe-associated molecular patterns and in intersections with effector-triggered immunity. Recent findings from yeast two-hybrid studies suggest that members of the TCP transcription factor family are targets of several effectors from diverse pathogens. Additional transcription factor families that are directly or indirectly involved in effector-triggered immunity are likely to be identified.

## INTRODUCTION

A common and early event in effector-triggered immunity (ETI) is the rapid up- or downregulation of pathogenesis-responsive genes. The advent of genomics and transcriptomics provided a comprehensive description of the magnitude of the transcriptional reprogramming that occurs in cells responding to detected effectors (Tao et al., 2003; Caldo et al., 2004; Adams-Phillips et al., 2008; Moscou et al., 2011). Subsequent findings of resistance proteins in the nucleus led to the suggestion that some resistance proteins directly affect transcriptional changes. A few well-discussed examples exist, but it is also clear that this proposed nuclear role is not a general feature of all resistance proteins. Interestingly, transcriptomics studies also highlighted the fact that transcriptional responses to avirulent and virulent pathogens mainly differ quantitatively (in the speed and amplitude of transcriptional changes), not qualitatively (in the identity of regulated genes; Tao et al., 2003; Katagiri and Tsuda, 2010). The layered nature of the plant innate immune system, where ETI is layered on top of the pathogen-associated molecular pattern-triggered immunity (PTI) network, makes it difficult to distinguish between genuine ETI-specific signaling steps, the guarding of PTI nodes by resistance proteins, and an accelerator function of resistance proteins that speeds up and amplifies an underlying PTI response (Shen et al., 2007; Gassmann and Bhattacharjee, 2012). Here we briefly review existing evidence for and against a nuclear function of resistance proteins and other ETI-associated proteins, but mainly focus on gaps that need to be filled to understand how to connect resistance proteins to the vast transcriptional response observed during ETI.

From a pathogen’s perspective, ETI is an unintended consequence of deploying effector proteins to colonize a host (Dangl and Jones, 2001; Jones and Dangl, 2006; Dodds and Rathjen, 2010). Effector proteins evolved to increase the fitness of a pathogen on its host by modulating host physiology in a variety of ways. Some examples of diverse effector functions include modifying components of the immune system to evade detection (Block and Alfano, 2011), and redirecting nutrients to the apoplast to support pathogen growth (Chen et al., 2010). Detection of these effectors by resistance proteins can occur when resistance proteins directly bind cognate effectors, or indirectly when resistance proteins detect changes to an associated host protein brought about by effectors (van der Biezen and Jones, 1998). In terms of their virulence function, one might postulate that the most potent effectors would target transcription architectures that regulate defense genes. However, in a recent comprehensive screening, transcriptional regulators are under-represented in the identified hubs targeted by multiple effectors from two different pathogens, *Pseudomonas syringae* and *Hyaloperonospora arabidopsidis *(Mukhtar et al., 2011). This deficiency may be caused by a general under-representation in the libraries screened or by elimination from consideration of auto-activating transcription factors and chromatin-associated components in yeast two-hybrid assays. In biological terms, this finding could also signify that the transcriptional response is a late event that is not a primary barrier to an invading pathogen, or more likely that a robust transcriptional network is not an ideal target for disruption (Tsuda et al., 2009).

## RESISTANCE PROTEINS AS DIRECT SIGNAL TRANSDUCERS

The activation of *Arabidopsis* resistance to *Ralstonia solanacearum-*resistant allele (RRS1-R) in the presence of the *Ralstonia solanacearum* effector *Pseudomonas* outer protein P2 (PopP2) was, until recently, considered a classic example of a system in which an activated resistance protein may directly stimulate ETI-related transcriptional changes (Deslandes et al., 2002). RRS1-R contains a WRKY transcription factor-like C-terminal domain. Native RRS1-R is unstable, and co-expression of PopP2 stabilizes nuclear RRS1-R (Deslandes et al., 2003; Tasset et al., 2010). However, subsequent findings showed that RRS1-R functions as a negative regulator of defense and that PopP2 acetyltransferase activity is required for RRS1-R activation, but not stabilization (Noutoshi et al., 2005; Tasset et al., 2010). This suggests that a yet to be identified PopP2 substrate or a protein interacting with activated RRS1-R functions in co-ordination with RRS1-R to mediate the majority of ETI gene modulations. Candidates include the resistance protein RPS4, which genetically was shown to function with *RRS1-R* in providing resistance to multiple pathogen effectors from diverse organisms (Birker et al., 2009; Narusaka et al., 2009, 2013), and ENHANCED DISEASE SUSCEPTIBILITY1 (EDS1), which was found to be in protein complexes with RPS4 and related resistance proteins (Bhattacharjee et al., 2011; Heidrich et al., 2011; see below).

A second example is the barley resistance protein Mildew locus A 10 (MLA10), which upon activation by powdery mildew effector Avr_A10_ interacts with WRKY1 and WRKY2 in the nucleus. Silencing of these WRKYs enhances resistance to both compatible and incompatible pathogens, suggesting that these WRKYs function as defense repressors (Shen et al., 2007). *Arabidopsis* WRKY18, WRKY40 and WRKY60, which have sequence homology to barley WRKY1/2, bind to promoter elements of the positive defense regulator *EDS1* and the jasmonate pathway repressor gene *JASMONATE-ZIM-DOMAIN PROTEIN8* (*JAZ8*) to repress their expression (Pandey et al., 2010). However, constitutive activation of defenses is not apparent in *wrky18 wrky40 wrky60 *mutants. Instead, up-regulated basal defense genes prime these plants for enhanced resistance toward both virulent and avirulent pathogens (Shen et al., 2007; Pandey et al., 2010). WRKYs that have recently been identified as positive regulators of defenses also affect both layers of immunity (Bhattarai et al., 2010; Gao et al., 2013).

The transcriptome alterations that characterize ETI likely involve specialized transcription factors that cue from activated resistance proteins and amplify an existing PTI response. A recent advancement in understanding MLA10-mediated immunity supports this model (Chang et al., 2013). At resting state, MLA10 cannot interfere with the WRKY1 function to sequester the positive defense transcription factor MYB6. Upon activation, MLA10 not only abolishes WRKY1 repression of MYB6 but also potentiates the DNA-binding activity of MYB6. A remaining question is which transcription factor enables the reported conserved function of MLA1 in *Arabidopsis* (Maekawa et al., 2012), since *HvMYB6*-orthologous genes are likely limited to the grasses and are absent in *Arabidopsis* (Chang et al., 2013).

Padmanabhan et al. (2013) also recently reported that the tobacco resistance protein N, upon activation, acquires nuclear binding to SQUAMOSA PROMOTER BINDING PROTEIN-LIKE6 (SPL6). They demonstrated that SPL6 controls the expression of several defense genes such as *PR1* and *PAD4*, and is essential for TIR-NBS-LRR-triggered ETI. Interestingly, both MLA10 and N only interacted with MYB6 and SPL6, respectively, after activation, possibly reflecting conformational changes or oligomerization of resistance proteins as a prerequisite for these protein interactions (Chang et al., 2013; Padmanabhan et al., 2013). More recently, Panicle blast 1 (Pb1), a broad-spectrum rice resistance protein against *Magnaporthe oryzae*, was reported to interact with and stabilize nuclear-localized WRKY45 by inhibiting its ubiquitin-mediated degradation (Inoue et al., 2013). Knockdown plants in a susceptible background were unaffected in basal resistance against the blast fungus. For Pb1 it is not clear yet what the activation step is, since Pb1 possesses a degenerate NB domain that lacks a functional P-loop (Inoue et al., 2013). Nevertheless, these studies demonstrate direct induction of defense genes by resistance proteins via specific transcription factors.

## ONE SIZE DOES NOT FIT ALL: MULTIPLE PATHWAYS TO RESISTANCE

Resistance proteins are deployed where they can intercept effector functions. Plasma membrane localized RPM1 is activated upon sensing host-modification of RIN4 by the action of membrane-targeted effectors AvrRpm1 and AvrB (Nimchuk et al., 2000; Mackey et al., 2002; Liu et al., 2011). Nucleocytoplasmic N is activated upon sensing and interacting with the liberated chloroplast protein NRIP1 following perturbations by the tobacco mosaic virus effector p50 (Caplan et al., 2008). While a nuclear sub-pool of some resistance proteins are required for their immune functions (Burch-Smith et al., 2007; Shen et al., 2007; Wirthmueller et al., 2007; Cheng et al., 2009), RPM1 relocalization from the plasma membrane to the nucleus is not required to induce an ETI-response to AvrRpm1 (Gao et al., 2011). Therefore, nuclear signaling during ETI does not always involve activated resistance proteins as the sole carriers. This is also supported by the evolutionary evidence that, while several resistance-like proteins from other plant species like *Populus *(Tuskan et al., 2006) have domains resembling DNA-binding elements, most characterized *Arabidopsis* resistance proteins neither possess transcription factor-like domains nor have been generally identified as direct associates of transcription factors. Thus, nucleotide binding-leucine-rich repeat (NB-LRR) proteins did not evolve from transcriptional regulators. This conclusion may not be very surprising, since resistance-like proteins are increasingly being identified in defense-independent roles, not all of which directly relate to transcription (Faigón-Soverna et al., 2006; Kim et al., 2012). In addition, a small but measurable nuclear pool for many resistance proteins already exists at resting state, and the majority of these proteins remain cytoplasmic even after activation. Small changes in amounts of nuclear protein are therefore difficult to measure, and it has not been shown convincingly yet that resistance proteins relocate to the nucleus after activation. Nevertheless, within the confines of the nucleus even small changes in the number of protein molecules relative to the bulk protein in the cytoplasm, either by import or by preventing cycling out of the nucleus, may increase the concentration of nuclear protein considerably.

An in-depth understanding of immune signaling is also being formed by studies of the activated resistance-like protein SUPPRESSOR OF npr1-1, CONSTITUTIVE1 (SNC1) that is proposed to function by repressing transcription of negative regulators of defense (Johnson et al., 2013). Even though a *bona fide *avirulence gene recognized by wild-type *SNC1* has not been identified, it was shown that SNC1 exists in comparable protein complexes as the resistance proteins RPS4 and RPS6, and contributes to AvrRps4 recognition in the absence of *RPS4 *(Kim et al., 2010; Bhattacharjee et al., 2011). Genetic screens and subsequent molecular approaches on the auto-active mutant allele of *SNC1 *identified *TOPLESS* (*TPL*) gene family involvement, suggesting a nuclear function for activated *snc1*. TPL members function as transcriptional co-repressors in many plant signaling pathways (Pauwels et al., 2010; Krogan et al., 2012; Wang et al., 2012). The demonstration that a TPL family member, TOPLESS RELATED1 (TPR1), forms a complex with SNC1 leads to a model in which SNC1 interacts with TPR1 to recruit HISTONE DEACETYLASE 19 (HDA19) to remodel chromatin at promoters of negative defense regulators (Zhu et al., 2010). A recent large scale search for interactors of TPL members identified transcriptional regulators belonging to diverse families, suggesting a wide role of TPL members as co-repressors (Causier et al., 2012). Intriguingly, one of the identified members, TCP14, is a target of at least two unrelated pathogen effectors (Mukhtar et al., 2011). Members of the TCP transcription factor family regulate leaf morphology and have been recently implicated in hormonal signaling (Koyama et al., 2007; Danisman et al., 2012; Steiner et al., 2012). Interestingly, a TCP-family protein was reported to be involved in the activation of several *WRKY* genes in cotton (Hao et al., 2012). It is a common observation that uncontrolled induction of immunity compromises regular growth and development of plants (Alcazar et al., 2011). Whether TCPs are direct transcriptional mediators that contribute to this fine balance needs to be determined.

## VIRULENCE TARGETS AS CO-SIGNALING COMPONENTS OF ETI

A recent large protein interactome dataset identified multiple host targets that a given effector may act upon in its pursuit for virulence (Mukhtar et al., 2011). However, what is the *modus operandi* of an effector in this ever-expanding protein–protein interaction network of resistance-associated proteins? The *P. syringae* type III effectors are functionally versatile and may mediate processes as diverse as proteolytic processing, ubiquitination, or nucleotide transfer on host targets (Block and Alfano, 2011). These manipulations of host targets may play synergistic roles with activated resistance proteins toward transcriptional modulation during ETI. The *P. syringae* effectors AvrRps4 and HopA1 cause disruptions of EDS1 associations with their cognate resistance proteins RPS4 and RPS6, respectively, at a microsomal location (Bhattacharjee et al., 2011). The effector AvrRps4 is processed *in planta *(Sohn et al., 2009), and although it was deduced from transient overexpression studies in turnip that the processed C-terminal domain is sufficient for the triggering of ETI in *Arabidopsis*, two independent reports seem to suggest the potential of each of these processed AvrRps4 domains as interactors with EDS1 and an RPS4-containing complex (Bhattacharjee et al., 2011; Heidrich et al., 2011). The precise functions of these interactions require further experimentation to resolve the issue. The observation that EDS1 is enriched in the nucleus during ETI (García et al., 2010) may indicate that EDS1 liberated from tight molecular associations in the cytoplasm is a candidate transcriptional modulator. However, as seen with resistance proteins, forced nuclear enrichment of EDS1 alone does not trigger ETI. Therefore, biochemical functions of these unrelated effectors on EDS1 need to be identified.

Plasma membrane localized RPM1 and RPS2 resistance proteins guard RIN4, a common virulence target of the unrelated effectors AvrB, AvrRpm1, and AvrRpt2 (Nimchuk et al., 2000; Axtell and Staskawicz, 2003; Jin et al., 2003). The cysteine protease activity of AvrRpt2 cleaves RIN4 (Axtell et al., 2003; Axtell and Staskawicz, 2003; Mackey et al., 2003), whereas in the presence of AvrB or AvrRpm1 the host kinase RIPK phosphorylates RIN4 (Liu et al., 2011). These alterations of RIN4 trigger activation of the cognate resistance proteins RPS2 and RPM1, respectively. Since a nuclear pool of activated RPM1 is not necessary for function (Gao et al., 2011), other components of these systems are likely mediators for nuclear signaling. Indeed, Holt et al. (2002) identified the interaction of specific RPM1 domains with a DNA-binding protein, TIP49a. TIP49a functions as a negative regulator of plant defense, and mammalian orthologs of TIP49a are involved in transcriptional regulation (Kanemaki et al., 1997). The interaction between RPM1 and AtTIP49a is suggestive of a cytoplasmic sequestering of negative regulators by an activated resistance protein. The AvrRpt2/RPS2 system also identifies a putative component that may act in transcriptional reprogramming. Unlike the membrane-tethered native RIN4, the AvrRpt2-processed RIN4 fragments are soluble (Afzal et al., 2011). Whether these fragments translocate to the nucleus or remain cytoplasmic, and whether other host proteins that are substrates for AvrRpt2 protease function mediate gene induction regulation, requires further study. Perhaps strengthening the above notion is the observation that modified RIN4 proteins which are deficient in plasma membrane binding constitutively activate ETI-type responses (Afzal et al., 2011).

Other post-translational modifications of proteins, for example through ubiquitination or SUMOylation, are likely to play a role in ETI as well. Ubiquitination has been observed to regulate resistance protein stability (Goritschnig et al., 2007; Tasset et al., 2010), and its roles in plant immunity have been reviewed recently (Cheng and Li, 2012; Furlan et al., 2012). The covalent attachment of SMALL UBIQUITIN-LIKE MODIFIER (SUMO) to a protein also affects its function (Mazur and van den Burg, 2012; Cubenas-Potts and Matunis, 2013). SUMOylation, predominantly a nuclear event, can also modulate activities of transcription factors, co-repressors such as the TPL family, and DNA-modifying components such as histones (Gill, 2005). Interestingly, a mutation in *Arabidopsis* *SIZ1*, which encodes an E3 SUMO ligase, induces constitutive salicylic acid (SA)-mediated defenses and confers enhanced resistance toward *P. syringae* DC3000 (Lee et al., 2007). Key proteins associated with innate immunity such as PAD4, EDS1, SAG101, and NPR1 contain putative SUMOylation motifs (Lee et al., 2007). Whether these proteins are real substrates for SUMO-modifications and whether the SUMOylation machinery is recruited in ETI remains to be determined. Multiple effectors from *Xanthomonas campestris pv. vesicatoria* such as XopD and AvrXv4 either possess de-sumoylation activities or cause a global decrease in the host SUMOylation profile (Hotson and Mudgett, 2004; Roden et al., 2004). This strongly suggests that SUMOylation regulates aspects of nuclear ETI signaling.

## CHROMATIN CHANGES AT IMMUNE-RELATED GENES

Post-translational modifications on core histones include methylation, acetylation, and phosphorylation (Fuchs et al., 2006; Pfluger and Wagner, 2007). The chromosomal environments these modifications create for ETI-responsive genes may determine the speed and amplitude of defense responses. Indeed, several chromatin-related proteins are often identified in plant defenses (Ma et al., 2011). Typical post-translational modifications mark nucleosome assemblies of defense regulators (Alvarez et al., 2010). Immune-responsive genes such as *WRKY* genes and *PR1* are maintained in a “ready” state via the extent of methylation status (tri-, in contrast to mono- or di-methylation) on histone H3 at lysine4 position (H3K4me3; Santos-Rosa et al., 2002). Although primed, actual transcription of these genes is regulated by specialized activators and repressors. For example, *ARABIDOPSIS TRITHORAX1* (*ATX1*) encodes a histone methyltransferase that directly affects the H3K4 methylation intensity of several *WRKY* promoters and governs the expression of several *TCP* transcription factor and NBS-LRR genes, including *CSA1* and *SNC1 *(Alvarez-Venegas et al., 2006, 2007). Interestingly, ATX1 is mostly cytoplasmic in un-elicited cells, suggesting that directed transcriptional re-programming during ETI may involve coordinated recruitment of specific histone methyltransferases and nuclear transcription factors. SET (Su(var)3-9, E(z) and trithoraxconserved) DOMAIN GROUP8 (SDG8), another histone methyltransferase, was recently reported to affect the H3K4me3 status-dependent expression of an *RPS4*-like resistance gene (Palma et al., 2010).

Histone acetylation and deacetylation modulate transcriptional efficiencies through activation and repression, respectively (Wang et al., 2010; Shakespear et al., 2011). A histone deacetylase (HDAC), REDUCED POTASSIUM DEPENDENCY3/HDAC1 from maize, confers resistance to the fungus *Cochliobolus *(*Helminthosporium*) *carbonum *through an unknown mechanism (Johal and Briggs, 1992). *Arabidopsis* AtHDAC19 has been identified to interact with several WRKYs and co-repressors (Zhou et al., 2005; Kim et al., 2008; Zhu et al., 2010). More recently, the *Arabidopsis* Elongator complex subunit 2 (ELP2), an active histone acetylase, was reported to influence the expression kinetics of *EDS1*, *PAD4*, and *PR1*, likely through the histone acetylation/methylation status (Wang et al., 2013). Further implication of histone acetylation in immune responses can also be extrapolated from the PopP2 acetyltransferase activity in RRS1-R elicitation (Tasset et al., 2010). Although this activity of PopP2, which may include histones as substrates, would likely aim to suppress defense, stabilized RRS1 as a result of the effector presence may hijack the mechanism to induce resistance-associated genes.

## CONCLUSION

**FIGURE 1:**
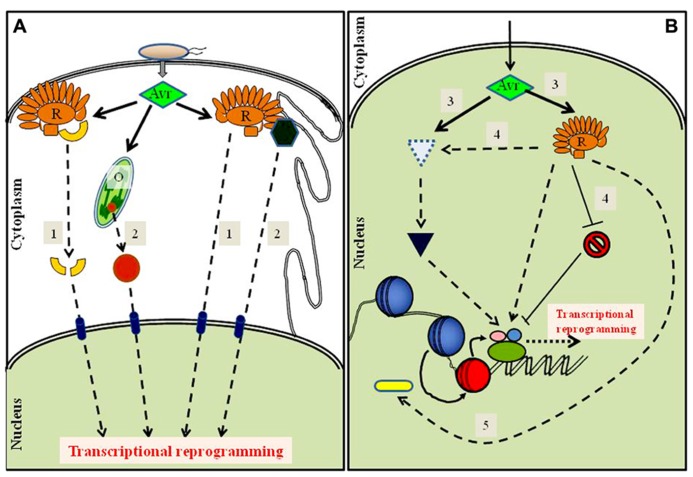
**Schematic diagram of possible cytoplasmic and nuclear routes to transcriptome reprogramming by an activated resistance protein.**> Detection of avirulence effector (Avr) presence or activities by a cognate resistance protein (R) may drive nuclear-directed signaling through multiple processes. **(A)** The cytoplasmic events may include, (1) direct nuclear translocation of effector-modified virulence targets or of the activated resistance protein itself, or (2) nuclear enrichment of a transcription-modulating protein sequestered in an organelle (O) or tethered to a membrane (e.g., ER). **(B)** Nuclear-targeted effector activities that trigger ETI may include, (3) promoting the stability of the sensing R protein itself or of a transcriptional activator, or (4) enabling an activated R protein either to sequester a negative regulator from or to recruit a positive regulator of defense to its target genes, or (5) altering chromatin by Avr- or R-mediated recruitment of chromatin remodeling components that further facilitate access by transcription factors. The strength and success of an effective ETI likely is determined by a tight co-ordination and possible synergism between some or all of the above processes.

An increasing amount of experimental evidence suggests a difference primarily in amplitude between PTI and ETI responses. To date, most identified modulators of transcription affect both branches of immunity, thereby clouding the interpretation of PTI- versus ETI-specific effects. Because in many cases effector activity and not simply the effector presence itself is the primary stimulus of ETI, an inherent deficiency of the routinely used yeast two-hybrid approach to identify resistance-associated proteins is the failure to incorporate this effector function. We have highlighted several potential areas where the function of an effector modulates the function of a host protein (**Figure 1**). Perhaps a more refined and directed approach is necessary in our search for transcriptional components. Stable lines expressing chemical-inducible effectors in susceptible and resistant hosts may provide one such PTI-independent system for proteomic approaches to identify differentially regulated nuclear proteins. In addition, genome-wide chromatin immunoprecipitation-sequencing (ChIP-seq)-based determination of transcriptional associations of activated NB-LRRs can be undertaken with this system. In parallel, precise biochemical functions of effectors need to be elucidated to understand host protein modifications. The vast interconnected ETI signaling web is clearly complex. Furthermore, any effector likely targets multiple host proteins. Whether robust and rapid ETI-associated transcriptome changes require synergistic signaling from different sectors or whether specific perturbations are direct transcriptional triggers needs to be elucidated. Transcriptional alterations require the coordinated actions of multiple DNA remodeling components, including specific transcription-associated proteins. Unraveling how nuclear signaling is achieved post-effector sensing and how this signal impinges on chromatin components is therefore necessary to understand and apply sustained resistance-developing technologies. 

## Conflict of Interest Statement

The authors declare that the research was conducted in the absence of any commercial or financial relationships that could be construed as a potential conflict of interest.
